# Self-defining memories in non-justice and justice-involved individuals: possible relations to recidivism

**DOI:** 10.3389/fpsyg.2023.1266392

**Published:** 2023-12-21

**Authors:** Hannah Elias, Elisa Krackow

**Affiliations:** Memory and the Law Lab, Department of Psychology, West Virginia University, Morgantown, WV, United States

**Keywords:** self-defining memories, memory, offenders, recidivism, risk assessment, autobiographical memory, identity

## Abstract

Given the high rates of recidivism in adults, additional efforts in this area are warranted. In this paper, we provide a developmental perspective on self-defining memories, a specific type of autobiographical memory. We review the literature on self-defining memories in offenders and non-offenders high in psychopathic traits. Next, we present an empirically based conceptual framework regarding self-defining memories and recidivism, including a model of recidivism that integrates self-defining memories with identity, decision making, and behavioral processes related to recidivism. We then critique this model. We call for future research to test this model. Should results be fruitful, we discuss potential applications of this work.

## 1 Introduction

Recidivism, an individual’s return to engagement in criminal behavior, is a problem that warrants substantial attention. A longitudinal study of recidivism in 24 states demonstrated that 68.8% of approximately 409,000 adult prisoners had been convicted of a crime by 10 years post-release, with the threshold exceeding 50% conviction at 4-years post-release (Antenangeli and Durose, 2021). In this paper, we integrate the literature on self-defining memories (autobiographical memories central to one’s identity) in justice-involved and non-justice involved individuals with literature on identity development, as well as literature on recidivism to present an integrated model of desistance, the manner by which people cease engagement in criminal behavior. Here we make clear that in doing so, we build on the work of others by combining the idea that self-defining memories should relate to recidivism (McLean et al., 2013) with the identity theory of desistance (Paternoster and Bushway, 2009) to present a model of recidivism that integrates this work. Taking a developmental perspective to document the origins and course of self-defining memories, we review literature on self-defining memories in non-justice and justice-involved individuals and make a case with novel components for why self-defining memories would be expected to differ in non-justice involved and justice-involved individuals. We then discuss how self-defining memories relate to identity and how self-defining memories and their components (e.g., insight) integrate with criminological theory and would be expected to relate to recidivism. Finally, we discuss clinical and forensic applications of this model with emphasis on autobiographical memories.

## 2 Autobiographical and self-defining memories

Developmentalists and personality psychologists conceptualize identity, the formation of the self-concept, in terms of autobiographical memories and narratives of those memories regarding the self (McAdams, 1988; Nelson, 2018). Identity development is of particular focus in adolescence and emerging adulthood, although continues to evolve throughout the lifespan (Erikson, 1968; Luyckx et al., 2006). That is, autobiographical narratives reflect memories of past events and help us understand the world and ourselves within the world (Nelson and Fivush, 2020). Autobiographical memories accumulate to ultimately make up a person’s life story (McAdams, 1988; Nelson, 2018). Narratives of these memories can be broader, as in an individual’s life story or narrower in terms of individual autobiographical memories of particular events, some of which might be described as self-defining memories (McAdams, 1988; Nelson, 2018).

Singer and Salovey (1993) first coined the term “self-defining memory,” to refer to those memories which are central to one’s identity (Singer and Salovey, 1993). These memories are emotional, vivid, and are associated with numerous other memories. They tend to be memories about “unresolved issues and enduring concerns” and serve as a window into a person’s thoughts, feeling, and personality (Singer and Salovey, 1993 p. 12). For example, clinicians may be able to abstract themes, such as abandonment, from a client’s self-defining memories. Self-defining memories retain their status in our lives because they are often tied to goal attainment or failure to attain goals, per empirical studies (Singer and Salovey, 1993). Important autobiographical memories, such as self-defining memories, can be kept private or made public, although sharing these memories through the initial telling and re-tellings helps people further conceptualize the self through making meaning of these events (Nelson, 2018).

Narratives of autobiographical memories also help people tie the past, present, and future together, thereby functioning to preserve a sense of self within and across developmental stages (Nelson, 2018; Jiang et al., 2020, for empirical evidence). For example, within developmental stages, the act of retrieving autobiographical memories increases the number of self-defining statements that people generate (Charlesworth et al., 2016) and self-concept influences preference for engaging in autobiographical recall (Jiang et al., 2020). Moreover, recalling positive autobiographical events in which goals were attained or negative memories in which goals were not attained can increase or decrease self-concept, as well as evoke positive or negative emotions (Singer, 1990; Conway and Pleydell-Pearce, 2000). Once constructed, narratives of self-defining memories can serve specific functions, such as to increase mood and guide decisions about future behavior (Singer, 2004). To build life stories, people add new (i.e., novel and re-interpreted) autobiographical memories that they perceive to be part of their identity and life themes. These additions to the life story are formed via “self-event connections” (Pasupathi et al., 2007). Although many memories meet these criteria, those that are highly relevant to one’s goals are strong candidates for becoming self-defining memories (Conway et al., 2004). Challenges within particular developmental stages are predictive of the types of strong memories that become self-defining memories (Conway et al., 2004). For example, emerging adults may have self-defining memories of leaving for college (theme of independence) and older adults’ self-defining memories may include losing a spouse (theme of identity).

Additionally, autobiographical memories allow people to look back at events from various perspectives of the characters present or as an observer which can enable us to “reflect on the actions of the past self and on the reactions of the social others in the scene or in the event” (Nelson, 2018, p. 269). What Nelson (2018) describes here is a prerequisite to meaning making, the process of effortful reflection that results in learning about the self (Thorne et al., 2004; McLean et al., 2013).

Personality can also influence the characteristics of self-defining memories in young adult college students. Positive emotionality, negative emotionality, and restraint relate most closely to three of the Big 5 personality characteristics (extraversion, neuroticism, and conscientiousness, respectively) to the contents of self-defining memories (Blagov and Singer, 2004; Blagov et al., 2021). Higher positive affect was associated with greater meaning making but higher negative affect being associated with lesser meaning making. Participants with the lowest levels of self-restraint were least likely to engage in meaning making, whereas participants with moderate levels of self-restraint were the most likely to engage in meaning making (Blagov and Singer, 2004; Blagov et al., 2021). Low memory specificity was related to reduced positive affect and high negative affect (Blagov and Singer, 2004; Blagov et al., 2021). Personality functioning was not related to meaning making in a sample of adolescents (*M*age = 19.5 years) hospitalized for mental health difficulties 6- or 12-months post-intake. However, self-defining memory valence was connected to personality functioning (i.e., identity, self-direction, empathy, intimacy), with positive valence related to better personality functioning and negative valence related to more compromised personality functioning (de Moor et al., 2022).

Importantly, culture can shape self-defining memories, including what events are remembered, as well as the content of memories and memories related to specific emotions (Ross and Wang, 2010; Wang and Singer, 2021). For example, the distribution of reported memories in which the main theme is relationships were similar amongst Chinese and American college students. In contrast, the percentage of guilt/shame themed self-defining memories was higher in Chinese college students compared to American students, and Chinese college students’ memories were much more likely to contain mention of academic stress (e.g., 23.3 vs. 3.33%) (Wang and Singer, 2021; Jiang et al., 2022).

## 3 Self-defining memories, meaning making, and identity

According to Habermas and Bluck (2000), the following cognitive abilities and skills are prerequisites to the formation of a life story; life stories tend to emerge in adolescence so that it reads life stories tend to be constructed beginning in adolescence. These include the following: (1) the ability to correctly sequence individual autobiographical event components and knowledge acquisition regarding the age/developmental period that particular life events generally occur (e.g., high school graduation tends to occur in late adolescence and college graduation in early adulthood); (2) understanding how the self can change but remain the same, including factors that are responsible for continuity (e.g., personality) and discontinuity (e.g., life events); and (3) the ability to describe oneself, including change in oneself. People must also possess the ability to abstract themes from stories, including those about others; to interpret stories; and to make inferences about (life) events to facilitate interpretation. According to Singer and Bluck (2001), (1) the above-described cognitive requirements allow people to determine which events are possible candidates for inclusion in the life story; (2) abstract life lessons from the life story; and (3) speculate about reasons particular life events may have occurred.

Given that self-defining memories are pieces of the life story, the above outlined cognitive advances required for the construction of the life story are also required to formulate self-defining memories (Singer and Bluck, 2001). One of the hallmark components of a self-defining memory is meaning making which can take two forms—lesson learning or gaining insight from the event depicted in the self-defining memory, with the difference being lesson learning would apply to similar future events but gaining insight would apply to a wider range of events (McLean and Thorne, 2003; Thorne et al., 2004).

Meaning making requires intentional reflective processing, as evidenced by relatively low levels of its presence in young adults’ self-defining memories (Thorne et al., 2004). For example, only 23% of 504 self-defining memories obtained from a sample of 168 undergraduate students contained meaning making via either insight or lesson (Thorne et al., 2004). Meaning making can be characterized as learning that is positive, negative, or a combination of positive and negative realizations about the experience (McLean et al., 2013). Empirical evidence demonstrates bi-directional influences between meaning making and identity in that meaning making not only influences identity, but identity can influence meaning making (McLean, 2005; McLean and Pratt, 2006). That is, the act of making meaning of one’s life increases knowledge of the self (identity) and people with stronger identities are better able to make meaning of life events (McLean, 2005; McLean and Pratt, 2006).

Before reviewing literature on self-defining memories of justice-involved individuals, we will now review literature on self-defining memories in adolescents and young adults not involved in the legal system, although we note that some of the about-to-be-reviewed studies include a broad age range of participants that stretch far into adulthood.

## 4 Self-defining memories from a lifespan perspective

Self-defining memories serve several functions for mid-aged adolescents and emerging adults (16–27-year-olds, mean age 18.7), most commonly to communicate about the self to others (27%), providing further evidence for the significance of self-defining memories in the identity development process (McLean, 2005). Less commonly, self-defining memories function to entertain others (17%) and to achieve intimacy via sharing memories, as well as function to validate the self and to make meaning of the event (all <10%, McLean, 2005). These memories often describe relevant development points at which certain events occurred (e.g., age of first romantic relationship) and concern relationships or achievements. The recipients of adolescents’ memory tellings are most often friends and parents, with friends becoming more common recipients as adolescents age (McLean, 2005).

Meaning making, including both lesson learning and gaining insight, begins to appear at age 14 in adolescents’ narratives of self-defining memories, such as of parent–child relationship conflict (McLean and Thorne, 2003), although we do note that some rudimentary levels of meaning making appear in some investigations of non-self-defining memories at ages 12 and 13 (McLean et al., 2010). Meaning making occurs equally in males and females (McLean, 2005).

Thorne et al. (2004) investigated the frequency of self-defining memories that included meaning making within particular event contexts. To do so, they asked undergraduate students to recount and include event details of three self-defining memories in written form. The authors then coded these memories by life event type (relationship event, mortality event, achievement event, and leisure event), the presence or absence of tension, and the presence or absence of meaning as reflected by insight or lesson. Overall, 23% of memories contained meaning via either insight or lesson. The relationship and mortality memory categories contained the highest prevalence of meaning (29 and 27%, respectively). Significantly, the low levels of meaning making demonstrated in this investigation show that meaning making does not occur without intent, nor is it the predominant response to a life event.

In McLean’s (2005) sample of 16–27-year-olds (mean age 18.7, *SD* = 1.2 years), the rate of meaning making was 31%, for which the breakdown was 10% lessons learned and 21%, insight. This was slightly higher than in the above-mentioned Thorne et al. (2004) study. Similarly, when meaning making was present in college students, whether positive or negative, the average ratings were <2 (2 = “minimal meaning making”) in each of 3 studies reported in McLean et al. (2018). Similar levels of meaning making were found in McLean and Pratt’s (2006) investigation in which youth were studied longitudinally beginning at age 17 and again at ages 19, and 23. Moreover, lesson learning and gaining insight occurred equally in adolescent’s narratives of self-defining memories regarding how their lives are consistent with and deviate from majority societal norms and behaviors (18 and 21%, respectively) (McLean et al., 2018).

Meaning making, particularly of negative events, tends to be beneficial (McLean and Pratt, 2006; Pals, 2006) and may serve the function of enhancing wellbeing. In young adults, distress was high for those college students whose negative self-defining memories lacked meaning making, but not for those whose self-defining memories included meaning making, thereby demonstrating that meaning making reduces the likelihood of distress (Blagov et al., 2021). However, there are exceptions to meaning making yielding positive outcomes (see McLean et al., 2010; Lilgendahl et al., 2013, younger adolescents only; de Moor et al., 2022 hospitalized adolescents with severe psychopathology).

Is meaning making associated with identity? McLean and Pratt (2006) examined meaning making at age 23 in turning point narratives, descriptions of events regarding either life transitions or in which substantial modifications occur to the self. Their goal was to determine whether meaning making was associated with Marcia’s (1966, 1967) stages of identity development. Marcia’s stages vary with regard to the dimensions of exploration and commitment. Some stages capture greater self-exploration by an individual, and finality of decision making with regarding major arenas of life (e.g., career); other stages capturing combinations of the absence of exploration and commitment (Marcia, 1966, 1967). Indeed, individuals with more advanced identity development at the ages of 17, 19, and 23 engaged in greater levels of meaning making at age 23. Moreover, meaning making can relate to one’s moral identity. In a sample of ages 18–75 (ages 27 and 51–59 excluded), lesson learning displayed in narratives on the topics of a moral incidence central to one’s conceptualization of morals and a narrative intended to educate an adolescent about honesty, correlated significantly with levels of moral reasoning and generativity concern, but did not correlate significantly with age (Pratt et al., 1999).

With regard to whether meaning making continues to advance across the lifespan, findings from a study comparing self-defining memories in younger and older adults demonstrate that indeed meaning making continues to advance as people mature. Older adults display higher percentages of meaning making but lower memory specificity than do younger adults (Singer et al., 2007). The topic distributions of the self-defining memories (i.e., relationship content, achievements, and life-threatening events) do not differ across college students and older adults (age range 50–85) (Singer et al., 2007). Older adults provide self-defining memories that reflect more positive emotions and less negative emotions, neither of which are not attributable to increased subjective well-being that typically occurs in older adulthood.

Another important aspect of self-defining memories is their level of detail (i.e., specificity) (Blagov and Singer, 2004). When memories do not include a description of a specific event, they are referred to as overgeneral memories (Williams, 2006). From a developmental perspective, even preschoolers are able to provide specific memories when tested with the Autobiographical Memory Test-Preschool Version (Lawson et al., 2020). Overgeneral recall can be attributed to rumination (high), avoidance (high), and executive control (lower), per the CaRFAX model [i.e., Capture and Rumination, Functional Avoidance, and Executive control (X)] (Williams, 2006).

In summary, self-defining memories serve several functions, including aiding identity development and serving the social functions of providing a mechanism for people to communicate about themselves to others and to entertain others (McLean, 2005; McLean and Pratt, 2006; Nelson, 2018) and may function to enhance wellbeing. Self-defining memories can contain not only event details, but also the lessons people learn from the event and how this insight might generalize to other events (Thorne et al., 2004). These lessons and insight are referred to as *meaning making* in the literature (McLean and Thorne, 2003). Meaning making begins to occur at age 14 (McLean and Thorne, 2003) and continues to develop into older adulthood (Singer et al., 2007). Meaning making is not automatic; it requires intent and occurs relatively infrequently (approximately 25–30%) in narratives of memories (Thorne et al., 2004; McLean, 2005; McLean et al., 2018). Meaning making also requires a particular level of memory specificity. Without recall of specific details of self-defining memories, meaning making is not possible.

## 5 Self-defining memories in offenders and non-offenders high in psychopathic traits

We will now review a series of studies that investigate self-defining memories in justice-involved individuals, a number of which compare self-defining memories in offenders to control groups of non-offenders, but before doing so, we will elucidate numerous reasons why self-defining memories may differ across groups. Here we note that the majority of extant studies include adult populations.

Antisocial personality disorder is often diagnosed in offenders (Rotter et al., 2002). Compared to participants without a diagnosis of antisocial personality disorder, Lavallee et al. (2020) reasoned that participants with a diagnosis of antisocial personality disorder (ASPD) would exhibit lower levels of specificity and meaning making due to the high comorbidity of ASPD and trauma history. Lavallee et al. (2020) documented the tendency for people with histories of child maltreatment to exhibit increased likelihood of overgeneral recall and decreased likelihood of meaning making.

Both memory specificity and meaning making may be reduced in people with ASPD or conduct disorder, a diagnosis in which youth engage in antisocial behavior, given the widely documented attention and lack of impulse control deficits characteristic of those with a diagnosis of ASPD and conduct disorder (see Moffitt, 1993; Chamberlain et al., 2016 for review of these deficits; see DSM-5-TR™ for current diagnostic criteria, American Psychiatric Association, 2022). Blagov and Singer (2004) found that undergraduates who scored as having low or high levels of self-restraint (a variable comprised of subscales of “Suppression of Aggression, Impulse Control, Consideration of Others, and Responsibility,” p. 490–491) based on scores a pencil- and paper-measure of adjustment, recalled self-defining memories comprised of less meaning making than those with moderate levels of self-restraint. Interestingly, low levels of self-restraint as conceptualized in this measure overlap with antisocial personality disorder/conduct disorder. Both attention control and impulse control are necessary to think introspectively and analytically in order to make meaning of past experiences. Private speech (i.e., silent self-talk), which is necessary for behavior control, is absent in individuals with severe enough attention control/impulse control deficits that are found in individuals with ADHD (Barkley, 1997). In addition, people with an ASPD diagnosis do not recognize that the behaviors they perpetrate against others are in fact harmful to other people (Marsh and Cardinale, 2012, 2014). Therefore, people with an ASPD diagnosis may be less likely to reflect on events in general, which is necessary for meaning making.

Lavallee et al. (2020) compared a sample of Belgium adult males sentenced to a forensic hospital with an assigned diagnosis of ASPD (*n* = 22, mean age approximately 41 years) to a community comparison group. Comparison participants (*n* = 22) were recruited via social media advertisements and did not meet diagnostic criteria for antisocial personality disorder diagnosis nor any other psychiatric disorder. Recruitment of these samples allowed the authors to determine whether persons with a diagnosis of ASPD would exhibit difficulty with meaning-making and lower memory specificity, and whether the distribution of themes of the self-defining memories would differ compared to control participants.

Lavallee et al. (2020) asked participants to provide five self-defining memories from any point in their life span. Participants with an ASPD diagnosis included meaning making in only 7.5% of memories, which was a significantly lower percentage than the control sample (28.2%). Compared to control participants, participants with an ASPD diagnosis also recalled fewer highly specific self-defining memories (i.e., memories of a particular event that lasted fewer than 24 h) and more self-defining memories that were less specific, thereby combining general events with some specific recall. Finally, participants with an ASPD diagnosis recalled fewer self-defining memories that featured a theme of achievement compared to control participants, but there were no significant differences in the percentages of memories that the authors classified as belonging to the following theme categories: Life-threatening, Recreation, Relationship, Guilt/Shame/Moral, and Drug, Alcohol, Tobacco use.

A series of two investigations examined autobiographical memory specificity in offenders (Neves and Pinho, 2016, 2018). Male and female incarcerated offenders (ages 20–49 years, mean age 33.8 years, *SD* = 6.2) were compared to a sample of similar aged community participants (Neves and Pinho, 2016). The offender and community samples (*M* ages 34.4 and 33.7 years, respectively) were both composed of 59 participants, each with approximately equal numbers of males and females from Portugal whose ages ranged from 19 to 52 (Neves and Pinho, 2018). Both investigations excluded people with diagnoses of psychiatric disorders, but groups were equivalent in age, socioeconomic backgrounds, and levels of education.

In the 2016 investigation, researchers asked participants to recall one positive and one negative “personally important autobiographical memory” (p. 674) from each developmental period of childhood, adolescence, adulthood, and one recent memory from the previous month. The researchers articulated the ages that corresponded to each of these developmental periods. Researchers presented up to three cues for each of the eight developmental period/valence combinations (e.g., adolescence/positive memory) to prompt recall. In the 2018 investigation, participants completed the Portuguese version of the Autobiographical Memory Test in which a researcher presented participants with ten cue words, alternating between positively (e.g., fun) and negatively (e.g., danger) valenced words. The participants were asked to recall a specific autobiographical memory consistent with each cue that spanned no longer than 24 h. The authors reported that they asked participants to recall “personally important events that were not recent” (p. 93) meaning that the to-be-recalled events had taken place at least 1 year prior. Participants were allotted a 60-s time limit for recall. If the participant responded with a general memory, they were prompted to report a specific memory.

Both studies included the administration of measures of executive functioning following memory production (investigation 1, verbal fluency; investigation 2, three measures of executive functioning—verbal fluency, Mazes, Stroop test). Memories were scored using more lenient and strict criteria and scores were summed. Both investigations included participant ratings of their memories on a limited number of phenomenological variables.

Findings generally converged across investigations. Therefore, unless mentioned, results apply to both investigations. Offenders and controls ultimately recalled an equivalent number of positive and negative autobiographical memories (2016, 2018), but compared to controls, offenders required more external support to recall those memories in the form of experimenter-provided cues to elicit the positive memories and fewer cues to elicit the negative memories (2016). Offenders’ positive but not negative autobiographical memories were less specific than those of non-offenders (2016, 2018). Males’ recall of self-defining-like memories was less specific than females’ recall (2016). The authors’ suggested that negative autobiographical memories may be more available in memory due to offenders exhibiting higher levels of negative mood, and therefore exhibited a tendency to recall (negative) events consistent with their mood (2016).

On the phenomenological characteristics of importance and emotional intensity evoked by the recall of the autobiographical memories, offenders rated their negative but not positive memories as being of greater importance and emotional intensity. The overall valence rating and rating of the representativeness of the recalled self-defining experience to the person’s childhood, adolescent, adulthood, or recent memory (i.e., whichever developmental period the memory fell into) were equivalent in offenders and controls.

Compared to controls, offenders scored lower on all measures of executive functioning—verbal fluency (2016, 2018), Mazes, and number of Stroop test errors. Executive functioning as measured by some tests, but not others, related to memory specificity depending on gender. In the 2016 investigation, verbal fluency related to positive but not negative autobiographical memory specificity for men but not women. This result did not replicate in the 2018 investigation. For women but not men, tests of executive functioning sometimes correlated negatively with specificity of positive autobiographical memories (i.e., Stroop test errors, Stroop test total scores, and Mazes total score, sudden ceasing of Mazes test – a behavior that reflected heightened perceptions and avoidance of test difficulty). The latter also correlated positively with negative autobiographical memories. The authors highlight this combination of difficulty on executive functioning tasks, including discontinuation in the face of difficult cognitive tasks as explaining a large percentage of the variance (37%) in specificity of women’s autobiographical memory for positively valenced events.

In another investigation of adult offenders, Taple et al. (2019) examined phenomenological memory characteristics of self-defining memories in three groups of males in Spain (mean age 30.8 years, age range 18–64) – incarcerated offenders with a mental health diagnosis, incarcerated offenders without a mental health diagnosis, and community controls. Participants had been most commonly diagnosed with personality disorder, followed by adjustment disorder, and then psychotic disorder.

The researchers had participants write one self-defining memory and then asked participants to write a second self-defining memory, although this time it was a self-defining memory that “defines your most aggressive, transgressive self, including your most criminal self.” (English translation from Spanish, p. 716). Following attainment of both self-defining memories, participants rated each memory on 10 phenomenological characteristics.

No significant differences occurred between the two incarcerated groups, but both incarcerated groups rated the current emotional intensity of their memories as being greater and provided higher ratings of the importance of implications of their self-defining memories, both with large effect sizes. Compared to the control condition, the incarcerated groups perceived their memories to be more negative and more detailed, as well as perceived themselves to be older than controls at the time of the remembered event, with large effect sizes emerging for valence and age. Memory clarity, feelings evoked by the memory at the time of the event, and sense of physical threat evoked by the event described in the memory were also significantly higher in the offender groups than control group, but the effect sizes were small. Repetition was also higher in the incarcerated groups which might be considered rumination (a piece of the CaRFAX model) at the higher levels. However, because the authors combined the ratings of both memories, it is unclear whether the results would hold for each self-defining memory if they were to be analyzed separately, or alternatively, if they would hold only for the aggressive memory. Therefore, on all phenomenological variables, there were significant differences between both offender groups and the control group.

Blagov et al. (2023) examined whether psychopathic traits (boldness, meanness, and disinhibition) could be identified in narratives of self-defining memories and if so, whether those traits related to self-defining memory specificity and meaning making. Undergraduates provided 10 self-defining memories and completed a pencil-and-paper measure of psychopathy characteristics. Researchers only analyzed the data from people who scored at the high and low extremes on the measure of psychopathic traits (*n* = 120). Blind coders coded the narratives for memory specificity, meaning making, and evidence of the three psychopathic traits. People who scored high in disinhibition and meanness provided less-specific memories, whereas those who scored low in meanness also exhibited lower levels of meaning making. Disinhibition was equivalent across the high and low psychopathy groups.

The final study to be discussed is the only study that we are aware of that examines meaning making and engagement/re-engagement in criminal acts. In a within-subjects design, McLean et al. (2013) examined whether meaning making and agency presence (i.e., acceptance of responsibility for actions) in narrative memories were associated with time since engagement in last risky behavior. The participants were 15–19-year-olds who attended an alternative high school. These youth self-described as having committed a variety of illegal behaviors, some of which had resulted in the youth’s arrest (12/37 youth). Participants then described three memories, including one self-defining memory, one positive memory that they recalled that could have occurred at any point in their lifespan, and “a time the participant went against his or her sense of who one is/what one believes” (p. 436). The youth completed a questionnaire that asked them to describe the “risky behaviors” (p. 437, i.e., primarily illegal behaviors) they enacted within the last 12 months (examples provided by the authors include stealing, consumption of alcohol and illegal drugs, and “using over the counter drugs for nonindicated use” p. 438) and the length of time since they carried out each risky behavior. The authors coded the narratives for evidence of agency and meaning making.

Although agency and meaning making correlated moderately (0.60), agency correlated significantly and positively with criminal desistance, although meaning making correlated significantly and positively with total number of risky behaviors in which the youth engaged. In contrast to expectations, meaning making and time to next instance of risky behavior were not significantly correlated, but greater meaning making was associated with engagement in a *greater* number of risky behaviors. As noted by the authors, the population that attends alternative schools typically has a high rate of exposure to adverse life events, such as maltreatment, domestic violence, and poverty. The enhanced negative emotions and cognitions elicited via the processing of these painful negative life events may have decreased behavioral inhibition, thereby resulting in increased risky behavior (McLean et al., 2013). However, the authors emphasize that meaning making is a continual process. With additional cognitive advances that occur in development, such as the ability to reconcile seemingly opposing pieces of information, and/or emotional advances that may occur, such as development of adaptive coping skills, meaning making may still predict desistance (McLean et al., 2013).

The next three paragraphs provide a summary of this section. Some investigations indicate that compared to community participants, both juvenile and adult offenders’ self-defining memories differed in a number of ways. Offenders experienced difficulty retrieving self-defining or self-defining-like autobiographical memories of a positive but not negative valence, but it is unknown whether inconsistent results are due to the inclusion of different autobiographical memory measures across studies or whether this result actually did not replicate (Neves and Pinho, 2016; Neves and Pinho, 2018 nonsignificant results). In some studies, offenders and controls recalled an equivalent number of positive and negative events (Neves and Pinho, 2016, 2018), but in other studies controls recalled a greater percentage of positive memories, whereas offenders recalled a greater percentage of negative memories (Lavallee et al., 2020).

Compared to controls, offenders’ memories were more specific for negative events but less specific for positive events (Neves and Pinho, 2016, 2018; Lavallee et al., 2020). Also, offenders or non-offenders high on the trait of meanness were less likely to make meaning of the events that comprised their self-defining memories (Lavallee et al., 2020; Blagov et al., 2023). Offenders displayed lower levels of executive functioning on tests of verbal fluency and executive functioning (Neves and Pinho, 2016, 2018). However, female offenders displayed a pattern of results in which executive functioning deficits were negatively associated with specific recall of positively valenced autobiographical memories and avoidance of uncomfortable experiences (i.e., discomfort elicited by difficulty with the tests of executive functioning, Neves and Pinho, 2018). These results are consistent with the CaRFAX model (Williams, 2006) that likens less memory specificity to executive functioning and avoidance of negative emotions. Group differences in phenomenological characteristics abound (Neves and Pinho, 2016; Taple et al., 2019), with some results differing for negative and positive self-defining-like memories (Neves and Pinho, 2016).

Results were relatively congruent regardless of whether the samples in these studies included (1) justice-involved adults diagnosed with ASPD (Lavallee et al., 2020); (2) a combined group of justice-involved adults that originally consisted of one group in which a personality disorder (presumably ASPD) predominated but other diagnoses were present and a second group of offenders without mental health diagnoses (Taple et al., 2019); (3) justice-involved offenders without psychiatric diagnoses (Neves and Pinho, 2016, 2018); (4) undergraduates scoring high or low on select characteristics that overlap with ASPD/conduct disorder (Blagov and Singer, 2004; Blagov et al., 2023) and (5) a group of adolescents attending an alternative school, presumably due to severe behavioral issues (McLean et al., 2013). This demonstrates that self-defining memories can be compromised in mid-aged adolescents and adults who demonstrate antisocial or noncompliant behavior that crosses a threshold severe enough to warrant placement in facilities that either serve or predominantly serve those with behavioral difficulties. Despite meaning making and agency being highly correlated in the sample of juveniles attending an alternative school, agency but not meaning making correlated with amount of time until next risky behavior/crime was committed (McLean et al., 2013). It is worth noting that the mechanism responsible for differences in self-defining memories between people with and without ASPD/offending tendencies is unknown. It could be a comorbid factor such as trauma or as previously discussed (Williams, 2006), executive functioning deficits.

## 6 Associations between self-defining memories and recidivism: a conceptual framework

As mentioned, reoffending rates are high in adults. Although a complete and detailed synthesis of the many factors that influence recidivism is beyond the scope of this article, these include, but are not limited to, basic resources in society (i.e., employment for individuals at least 27 years of age) and some neighborhood factors (e.g., concentrated disadvantage) for adults with moderate but not high risk of recidivism (Uggen, 2000; Jacobs and Skeem, 2021). They also include relationship factors, such as visitation while imprisoned and having a positive relationship with an individual’s probation officer (Mears et al., 2012; Chamberlain et al., 2018). Further, individual difference factors contribute to recidivism such as low levels of academic achievement, low cognitive ability including verbal intelligence or intellectual disability, and diagnosis of psychiatric disorders, especially antisocial personality disorder (Katsiyannis et al., 2008; Edens et al., 2015). Characteristics of treatment may influence recidivism (i.e., intensive treatment not focused on sanctions for youth high on psychopathy characteristics, Caldwell et al., 2006). More recently, algorithms have been used to predict recidivism but not without controversy (see Skeem and Lowenkamp, 2020, for discussion).

Meaning making, including learning lessons and developing insight, might be expected to influence future behavior via changing past behavior, especially decreasing undesirable behavior, including the performance of criminal acts (McLean et al., 2013; Lavallee et al., 2020). That is, meaning making may reduce recidivism (McLean et al., 2013; Lavallee et al., 2020). In fact, Inderbitzin (2006) conducted a qualitative study (sample size not mentioned) of males, ages 15–20, who resided in a single cottage in a maximum-security juvenile facility, specifically regarding the lesson they learned while incarcerated. The author characterized the youth as “violent offenders” (p. 12). Consistent with meaning making, Inderbitzin concluded, “Finally, their time in the institution offered these young men a chance to reflect on their lives and their place in the world. It gave them the opportunity to really think about who they were before their incarceration, who they were turning into during their time in confinement, and who they wanted to be when they got out and grew up. (p. 22). The author indicated that meaning making did not occur universally among the youth participants.

In fact, some aspects of meaning making are considered in the assessment of risk of recidivism. Insight appears as a scale of the Historical-Clinical Risk Management-20 Version 3 (HCR-20:V3, Douglas et al., 2013), a measure of risk of recidivism in adults. Douglas and Shaffer (2021) describe the HCR-20:V3 as follows: the HCR-20:V3 combines information gathered on 20 items from a clinical interview with the offender and review of records to assess risk of recidivism. The evaluator is charged with not only gathering information but figuring out the circumstances that lead the person to commit the crime, termed “meaning making” (p. 255). Once the evaluator obtains the information, they decide whether each risk factor is present or absent. Risk factors can also be deemed to be “possibly/partially present.” (p. 256). If the evaluator deems the risk factor to be present, they continue to evaluate whether the risk factor increases the risk for this person, in part by deciding whether the risk factor drove previous criminal activity, whether it negatively impacts decision-making with respect to violence, and whether it might drive the presence of other risk factors. The evaluator continues to take additional steps to make decisions about risk, but those are less relevant to the current manuscript and therefore will not be described. The insight scale assesses for “recent problems with insight” in the areas of “mental health problems, risk for violence, and need for treatment” (Douglas and Shaffer, 2021, p. 259).

The case example included in Douglas and Shaffer (2021) described a person who was hospitalized in a forensic hospital for charges of Assault Causing Bodily Harm and Failure to Comply with a Restraining Order. The evaluator gave the person a diagnosis of paranoid schizophrenia. The evaluator judged her risk for insight to be “partially present” and described a person who attributed the reason for the crime she committed to external sources (i.e., her boyfriend), thereby failing to recognize and take responsibility for the circumstances in which she became violence. She also underestimated her chance of engagement in future violence. However, she correctly recognized the need to take steps to manage her mental illness symptoms, acknowledging that doing so might help her avoid future episodes of violence. Douglas and Shaffer (2021) reviewed 8 studies comprised of 25 samples, many of which were forensic samples. The area under the curve (AUC) was significant in 21/25 of the samples with AUCs ranging from 0.68 to 0.91 which indicates that the HCR-20 V3 correctly predicted risk for violence, sometimes described by the authors of the original studies as “serious violence” or “imminent risk.”

Other research has linked insight to recidivism in juveniles ages 12–22 years (Mulder et al., 2010, 2012). Based on a sample of youth who were amongst the most severe of criminals in the Netherlands and therefore hospitalized in forensic facilities. Mulder et al. (2012) identified four subtypes of juvenile offenders based on statistical analyses – violent property offenders, property offenders, serious violent offenders, and sex offenders. The authors then examined whether risk factors for recidivism, including whether insight differed across the groups. They did in fact find that insight was one factor that differed across offender subtypes with the lowest levels of insight in violent property offenders (those who completed violent offenses during property offenses) and property offenders. Although the researchers did not examine insight as a predictor of recidivism, these two groups also had the highest levels of recidivism at the minimum 2-year follow-up point (mean 5.83 years) with 89 and 82% recidivism, respectively. A substantial portion of the recidivism in these two groups encompassed violent recidivism and a small percentage (6 and 5%, respectively) of offenders in these two groups committed sexual offenses, seemingly representing a branching out to sex crimes. Interestingly these percentages were a few percentage points above the sexual recidivism rates of sex offenders (3%). The authors note that the low levels of insight along with low conscience scores in these two groups of offenders are consistent with psychopathy, although acknowledged they did not measure psychopathy. In a second investigation (Mulder et al., 2010) that included the same participants on which the 2012 study was based, additional analyses showed problem insight to be related to recidivism at 2 or more years post-release (mean 5.83 years). In reporting these results, we do not wish to imply that meaning making and insight are identical, but rather that insight is necessary but not sufficient for meaning making.

Taken together, these findings converge to suggest that people incarcerated for criminal behavior are less able to learn lessons and gain insight via meaning making from the events of their lives as compared to community controls. This is particularly noteworthy given that self-defining memories are, by definition, memories of events that individuals defined as having shaped their lives. Therefore, justice-involved individuals are able to identify events that are meaningful to them but are not able to make meaning of these events. This extends to self-defining memories of their own aggressive behavior, which possibility were the same events that resulted in their justice-involvement.

**Figure 1:**
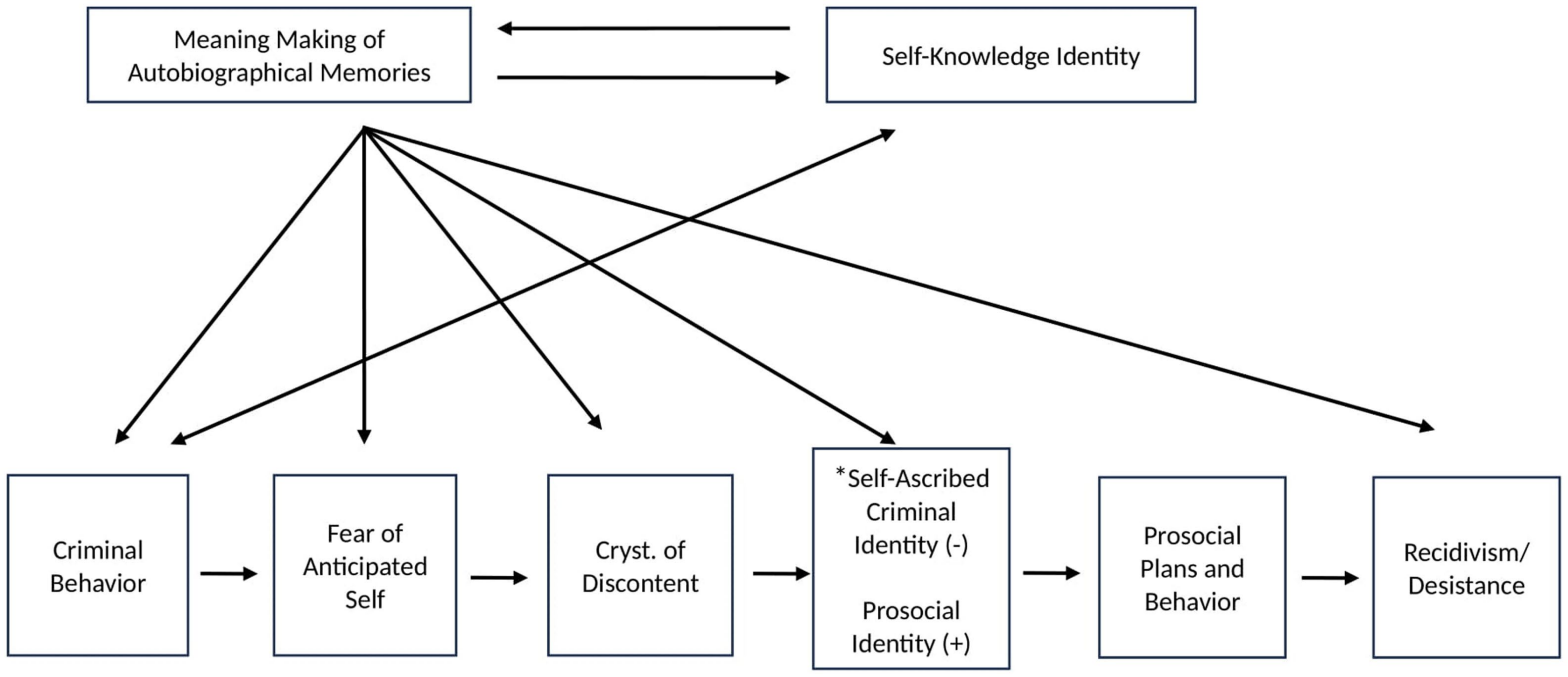
Conceptual model relating autobiographical memory meaning making, identity, and recidivism. Hypothesized associations between meaning making and identity which form the core processes of recidivism. Model combines the work of McLean (2005), McLean and Pratt (2006), McLean et al. (2013), and Paternoster and Bushway’s (2009) self-identity theory of desistance. ^*^Criminal identity must be relinquished in order to form prosocial identity.

In Figure 1, we outline a model of Paternoster and Bushway’s (2009) identity theory of desistance and acknowledge that this includes the combined work of McLean (2005), McLean and Pratt (2006), and McLean et al. (2013) along with the components of Paternoster and Bushway’s (2009) identity theory of desistance. We note here that Paternoster and Bushway (2009) take the position that at some point after a person engages in criminal behavior, perceptions of oneself change, such that the person ‘takes on’ that behavior as part of their identity. It is for this reason that we use the term “self-ascribed criminal identity.” We note that Paternoster and Bushway’s view differs from the more typical conceptualization in which criminal acts are viewed as behaviors, and thus remain external to the person, as opposed to becoming internalized.

We intend for this model to apply to adults, but at this point do not want to rule out the possibility that it could apply to mid-aged adolescents should future data become available that shows that adolescent meaning making is associated with recidivism. At this point, extant data (McLean et al., 2013) suggest that the model may not apply to adolescents. We now describe the model with non-obvious pathway names in brackets. According to Paternoster and Bushway’s (2009) identity theory of desistance, offenders must develop the desire to avoid a life of crime and its ensuing consequences which requires that the person first develop a fear of the consequences of future criminal behavior (i.e., fear of dying in prison, fear of not being able to spend time with loved ones; Liu and Bachman, 2021) [*criminal behavior → fear of anticipated self*]. The fear does not develop until the offender engages in multiple instances of legal involvement/recidivism, at which point the offender develops insight via associating their current situation with future consequences of continued engagement in criminal behavior [*criminal behavior → fear of anticipated self*; *meaning making → fear of anticipated self*]. This insight regarding their anticipated self serves as the catalyst for change. The person begins to recognize the negatives of criminal behavior, engages in a cost–benefit analysis of such behavior, and ultimately, becomes disenchanted with the criminal life. Paternoster and Bushway (2009) refer to the process of arriving at disenchantment as “the crystallization of discontent, part of a subjective process of self-interpretation or self-knowledge” (p. 99). To arrive at the discontent, the person goes through the process of meaning making as described here: “But a large pattern of problems and frustrations brings one up to a broader level of meaning and raises the issue of whether the positives outweigh the negatives. The person’s calculation of whether the involvement is worthwhile can no longer ignore the large body of problems (Paternoster and Bushway, 2009, p. 99).” [*meaning making → crystallization of discontent*]. The person then projects the representation of their self-ascribed criminal identity into the future, thereby envisioning their anticipated self if they do not change *[fear of anticipated self].* At this point the person decides to change their life trajectory. The cornerstone of the change process includes relinquishing their self-ascribed criminal identity along with any associated identities that they perceive to have played a causal role in their criminal behavior (e.g., “self-identity as an addict,” Liu and Bachman, 2021, p. 351) [*crystallization of discontent→ prosocial identity*]. In an attempt to change their life trajectory, the individual will engage in numerous positive behaviors to better themselves and society, for example, by obtaining further education (Paternoster and Bushway, 2009; Liu and Bachman, 2021) [*prosocial identity → prosocial plans and behavior*]. In addition, they will naturally disaffiliate from people associated with criminal behavior and form relationships with individuals who make prosocial contributions to society [*prosocial identity → prosocial plans and behavior*]. However, these change-inducing prosocial behaviors will not be effective if the relinquishment of the self-ascribed criminal identity does not occur [*prosocial identity → relinquishing of self-ascribed criminal identity → prosocial plans and behavior*]. Finally, after numerous setbacks in this process (i.e., re-engagements in criminal behavior), the person’s criminal behavior will drop off and eventually desistance may occur [*criminal behavior → recidivism/desistance*].

We note that Paternoster and Bushway’s (2009) theory overwhelmingly emphasizes identity change as the key mechanism for reducing criminal behavior, as indicated by their statement “identity change initiates desistance,” (Paternoster et al., 2015, p. 225) and the content of numerous quotations from people with criminal histories contained in the article on this theory (Na et al., 2015). Secondarily, their theory emphasizes the anticipated self. Although Paternoster and Bushway (2009) describe meaning making as the key process in “the crystallization of discontent” that leads to the desire to relinquish the self-ascribed criminal identity, as well as the identification of the anticipated self, we note that meaning making is ultimately de-emphasized. At best, meaning making takes a back seat to identity and at worst, it is nearly lost in the theory, so much so that it is barely present in affiliated authors’ (e.g., Liu and Bachman, 2021) descriptions of Paternoster and Bushway’s (2009) theory. We wish to bring meaning making to the forefront of the theory. We suggest that meaning making is central to this theory, although we do not make claims about its importance relative to identity. Hence, meaning making sets off a chain of events in which meaning making is central not only to the development of the anticipated self and the relinquishment of the self-ascribed criminal identity/development of the novel identity as a prosocial person but meaning making is also central to the development of the plan to engage in prosocial behavior. Therefore, meaning making is implied to be essential to desistance.

We also note that empirical evidence exists for the following components of the identity theory of desistance: reduction in criminal behavior is more likely to occur after multiple episodes of criminal behavior (Liu and Bachman, 2021) [i.e.*, criminal behavior → fear of anticipated self*]; Prosocial behaviors were unsuccessful in reducing criminal behavior if identity change had not occurred (Liu and Bachman, 2021) [*prosocial identity and recidivism/ desistance*]; identity change is prominent in desistance (Na et al., 2015) [*prosocial identity and desistance*]; juveniles are more likely to engage in ongoing criminal behavior if they identify as criminals (Na and Paternoster, 2019) [*criminal identity → criminal behavior*]. We now encourage researchers to specifically test aspects of the model that relate to meaning making such as whether meaning making predicts recidivism, using a longitudinal design. Next, we critique the model and present implications of the model focusing on how this could be applied in clinical-forensic contexts post-empirical investigation.

## 7 Discussion

### 7.1 Critique including limitations of the model

Paternoster and Bushway’s (2009) model, that focuses on change processes by outlining specific points of change leading to recidivism/desistance, is intriguing. Their model resonates with common sense. The model is based on literature, although sometimes based on conceptual as opposed to empirical contributions (Paternoster and Bushway, 2009). As previously noted, its focus is predominantly on identity as opposed to self-defining memories. However, we presuppose the model could be broadened. For example, Paterson and Bushman propose that people undergo a narrow life review specific to their criminal behavior, but we think it is possible that people engage in broad life review in which they analyze and attempt to make meaning making of their past experiences, including self-defining non-criminal and criminal-related autobiographical memories of life events. Some evidence for our view is provided by Inderbitzin (2006). Their model is unidirectional, but we suspect that there are points in which bi-directional influences are at force. For example, engagement in prosocial planned behaviors would presumably lead to post-event processing regarding whether the planned behaviors worked, did not work, or alternatively, which aspects worked/did not work (i.e., meaning making of the plans/behaviors). We further presuppose that engagement in prosocial behavior and post-event processing (i.e., meaning making) would in turn further strengthen one’s “prosocial identity” (Paternoster and Bushway, 2009, p. 1129), as well as increase one’s general identity (i.e., self-knowledge identity). Finally, engagement in prosocial behavior and post-event processing may facilitate reflection on the self-defining events of one’s life and lead to the attainment of new insights into those events (i.e., bi-directional influences between prosocial plans and behavior and meaning making of autobiographical memories). An additional limitation of the model is that per Paternoster and Bushway (2009), their model is meant to explain recidivism following repeated instances of criminal behavior and punishment, and therefore would not be expected to pertain to change in pathway earlier in the cycle of criminal behavior, which they describe as being less common.

### 7.2 Applications of the model

Looking forward post-empirical investigation, should sufficient evidence emerge that meaning making in self-defining memories is associated with reduced recidivism in justice-involved individuals, a forensic assessment that includes a self-defining memory recall task (Thorne et al., 2004; McLean et al., 2013) could be undertaken. However, the first measure of the assessment should be one that assesses memory specificity such as the Autobiographical Memory Test given that meaning making is unlikely to occur without memory specificity. If the examinee performs sufficiently on the Autobiographical Memory Test, then the self-defining memory task would be undertaken. During the self-defining memory task, individuals could be asked to recall several self-defining memories (as in Thorne et al., 2004). If during the recall task, the examinee does not include the event that resulted in their current justice-involvement as a self-defining memory, a separate prompt could be added to elicit recall of that event (in the spirit of Taple et al., 2019). These memories could then be coded for meaning making and specificity using existing coding systems. Note, here the ability to make meaning in non-criminal self-defining memories would be considered to be as important as making meaning of criminal self-defining memories, unless future research results were to suggest otherwise. Also important would be the measurement of criminal identity. The assessment results, in conjunction with other empirically supported factors, could be used to assist in a determination as to whether the individual can be released.

If there is not sufficient specificity within the examinee’s recall of their self-defining memories, efforts to increase specificity of self-defining memories could be undertaken given that meaning making requires sufficient knowledge of the details of the to-be-remembered events. Memory specificity training, an intervention in which individuals are instructed how to recall memories in a specific fashion, is effective (per meta-analysis, *d* = −1.21) for adolescents and adults with depressive symptoms (Barry et al., 2019). Following intervention, the Autobiographical Memory Test could be re-administered and if memory specificity is sufficient the self-defining memory task could be administered at that point.

Even if a person is able to make meaning of events related to non-justice involvement and justice-involvement and because of this might seem to be at low risk for recidivism, intervention for antisocial behavior should be considered. Where possible, we recommend interventions that are developmentally appropriate, empirically supported, and specifically designed to reduce antisocial behavior in conjunction with psychotherapies based on the processing of negative self-defining memories.

Psychotherapies in which self-defining memories are the main target of the intervention can be used to help clients gain insight into how these life events influence current behavior (Singer and Blagov, 2004). For example, Singer and Blagov (2004) and Singer et al. (2013), articulate client examples in which wellbeing can be enhanced by working with the client’s repeated life themes (narrative script) articulated in therapy. Here the therapist uses techniques to change the theme and behavior associated with the theme by decreasing avoidance of negative emotion. This enables the client to gain memory specificity and engage in meaning making, which in turn decreases psychological symptoms and/or improves interpersonal functioning. Finally, Çili et al. (2017) used imagery rescripting to decrease distress regarding negative self-defining memories. Following a 3-session intervention in which participants viewed the negative childhood events from the perspective of themselves today as adults and provided feedback to their childhood self to allay the negativity. In one session, the students “took the perspective of their younger self” (p. 82) and had other characters provide emotional support to their childhood self. Following this final portion of the intervention, a number of memory characteristics had changed—most notably, distress following recall (large effect size), memory negativity, and perceptions of how much the memory importance to their current view of themselves with medium effect sizes. The authors note the inclusion of a college student sample, as opposed to a clinical sample, and the lack of a control group as design limitations, although several participants had past psychiatric diagnoses.

In closing, we make the case that meaning making of autobiographical events is central to the development of identity, and that self-defining memories in justice-involved individuals are different than in non-justice involved individuals. We present a model and critique of the model in which the ability to make meaning of a wide range of autobiographical memories of events, including but not limited to criminally related events, is critical to the process of desistance. The role of meaning making in desistance is an important yet relatively unexplored area of research. We call for researchers to test this model, either in parts or in its entirety. If after post-empirical investigation and replication, sufficient evidence was to emerge that meaning making and specificity of self-defining memories, (both unrelated to and regarding the most recent crime that resulted in current justice-involvement) relates to recidivism, we propose that self-defining memory features and identity be considered be as factors in readiness for release from incarceration. Even if an individual is considered to be ready to be released, we encourage professions to consider the possible need for evidence-based treatments in helping to reduce the chances of recidivism and improve the quality of life.

## Author contributions

HE: Conceptualization, Writing – original draft, Writing – review & editing, Visualization. EK: Conceptualization, Writing – original draft, Writing – review & editing, Visualization.
